# Antifouling Conductive Composite Membrane with Reversible Wettability for Wastewater Treatment

**DOI:** 10.3390/membranes12060626

**Published:** 2022-06-16

**Authors:** Yi Li, Ruonan Gao, Jianwen Zhang, Yue Zhang, Shuai Liang

**Affiliations:** 1 Beijing Key Lab for Source Control Technology of Water Pollution, College of Environmental Science and Engineering, Beijing Forestry University, Beijing 100083, China; liyi0421_bjfu@163.com (Y.L.); gaoruonan0416@163.com (R.G.); zjw3200412@bjfu.edu.cn (J.Z.); yuer_z@bjfu.edu.cn (Y.Z.); 2 Engineering Research Center for Water Pollution Source Control & Eco-Remediation, College of Environmental Science and Engineering, Beijing Forestry University, Beijing 100083, China

**Keywords:** conductive membrane, antifouling, smart membrane, electro-responsive, reversible wettability

## Abstract

Membrane fouling severely hinders the sustainable development of membrane separation technology. Membrane wetting property is one of the most important factors dominating the development of membrane fouling. Theoretically, a hydrophilic membrane is expected to be more resistant to fouling during filtration, while a hydrophobic membrane with low surface energy is more advantageous during membrane cleaning. However, conventional membrane materials do not possess the capability to change their wettability on demand. In this study, a stainless steel mesh–sulfosuccinate-doped polypyrrole composite membrane (SSM/PPY(AOT)) was prepared. By applying a negative or positive potential, the surface wettability of the membrane can be switched between hydrophilic and relatively hydrophobic states. Systematic characterizations and a series of filtration experiments were carried out. In the reduction state, the sulfonic acid groups of AOT were more exposed to the membrane surface, rendering the surface more hydrophilic. The fouling filtration experiments verified that the membrane is more resistant to fouling in the hydrophilic state during filtration and easier to clean in the hydrophobic state during membrane cleaning. Furthermore, Ca^2+^ and Mg^2+^ could complex with foulants, aggravating membrane fouling. Overall, this study demonstrates the importance of wettability switching in membrane filtration and suggests promising applications of the SSM/PPY(AOT) membrane.

## 1. Introduction

Water scarcity and water environment pollution have been severely hindering the sustainable development of human society. Wastewater reclamation is an important strategy for alleviating the water crisis. Membrane separation technology (including microfiltration, ultrafiltration, nanofiltration, reverse osmosis, etc.) has become one of the leading technologies for wastewater reclamation [1,2,3]. However, the membrane fouling problem has seriously restricted the further development of membrane separation technology [4,5]. The mitigation of membrane fouling has been the research focus in the membrane separation area in recent decades [6,7,8].

Controlling the physical and chemical properties of membrane materials is an effective way to mitigate the occurrence and development of membrane fouling [9,10,11,12]. The physical and chemical properties of membrane surfaces mainly include membrane porosity, wettability, surface charge, and so on [13]. In recent years, it has been widely reported that membrane wettability is a dominant factor that affects membrane fouling behavior [14,15]. Great efforts have been made to modify membrane surface wettability. A series of membrane modification strategies, such as blending and grafting, have been developed [15,16,17]. However, the inherently high surface energy of hydrophilic membranes may lead to the firm adhesion of foulants on their surfaces. Therefore, in the membrane cleaning stage, high surface energy is not conducive to the removal of foulants, thus reducing cleaning efficiency and increasing the risk of irreversible membrane fouling. In contrast, the foulants on hydrophobic surfaces with low surface energies are more likely to be rinsed off in the membrane cleaning stage [18]. Accordingly, membranes that are hydrophilic during filtration but hydrophobic during cleaning may exhibit superior resistance to membrane fouling. However, conventional membrane materials do not possess the capability to change their wettability according to different operational demands.

With the ongoing development of materials science, smart responsive materials that can adjust surface wettability under certain stimulations, such as temperature [19], pH [20], light [21], electric [22], and magnetic field stimulations [23], have been developed [24,25,26]. These materials can be used to further improve membrane separation performance. In recent years, electro-responsive membranes have gained much attention due to the rapid and convenient application of electric potential on conductive membrane surfaces. In a wide range of applications, electro-responsive membranes have been used, for example, in sensors, electronic devices, and bionic devices [27,28,29]. Typically, conductive polymers have conjugated main chains containing series of alternating single and double carbon bonds that allow electrons to delocalize and move freely between atoms [30,31]. In the synthesis of conductive polymers, various dopants (in most cases, with negative charges/anions) can be introduced into the polymers. When the applied voltage is adjusted, the doping level (oxidation level) of the conductive polymer changes accordingly, resulting in changes in membrane properties, such as pore size, color, surface wettability, and so forth [32].

Conductive polymers commonly used for electro-responsive membranes include polypyrrole (PPY), polyaniline, polythiophene, poly (3,4-ethyldioxypyrrole), and so forth [33,34,35,36]. However, membranes made of conductive polymers alone usually have weak mechanical strength and consequently poor filtration performance. In contrast, composite conductive membranes made by combining conductive polymers with other materials via in situ electro-polymerization usually have better conductivity, stronger mechanical strength, and better filtration performance, suggesting a greater potential for practical applications. Currently, however, studies on conductive composite membranes with electro-responsively reversible wettability for municipal wastewater treatment are rarely reported. Their fouling behaviors and corresponding operating strategies need to be systematically investigated.

In this study, a conductive stainless steel mesh/sulfosuccinate doped PPY (SSM/PPY(AOT)) composite membrane with reversible wettability was prepared for municipal wastewater treatment. Systematic characterizations and a series of filtration experiments were carried out. The fouling behavior of the membrane in four different operating modes was investigated.

## 2. Materials and Methods

### 2.1. Preparation of the SSM/PPY(AOT) Composite Membrane

**Figure 1:**
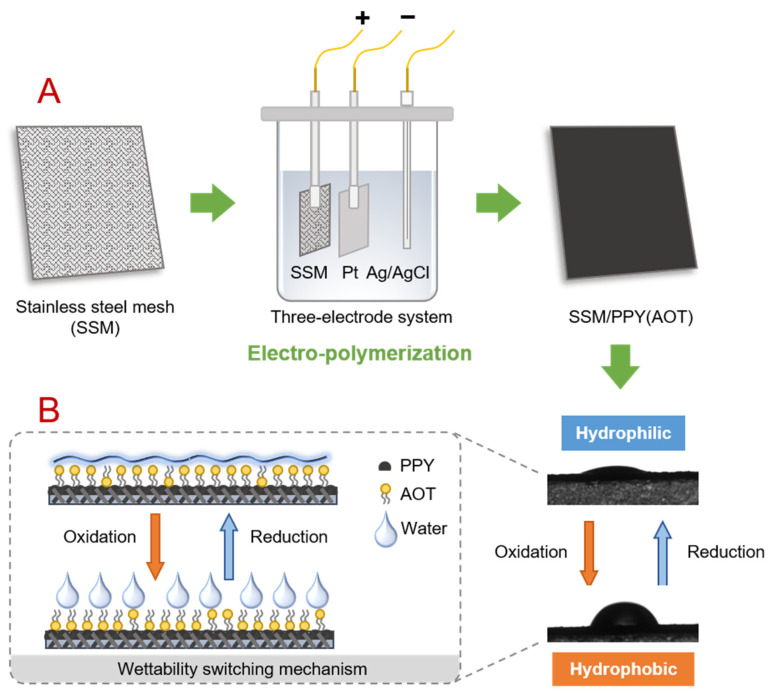
(**A**) Schematic protocol for the preparation of the SSM/PPY(AOT) membrane via electro-polymerization. (**B**) Illustration of the wettability switching behavior and the mechanism of the SSM/PPY(AOT) membrane.

The SSM/PPY(AOT) membrane was prepared by implementing an in situ electro-polymerization strategy (Figure 1) using pyrrole (99%, Macklin, Shanghai, China), sodium bis(2-ethylhexyl) sulfosuccinate (commonly abbreviated as AOT; 96%, Macklin, China), and SSM (Anping Metal Ltd., Anping, China) as polymeric monomer, dopant, and base material, respectively. The pyrrole was pre-purified by vacuum distillation and stored at 4 °C prior to use.

For electro-polymerization, the SSM (working electrode), platinum plate (counter electrode), and Ag/AgCl electrode (reference electrode) were immersed in an electrolyte solution (Figure 1) which contained 0.30 M pyrrole as monomer, 0.1 M AOT as dopant, and 0.2 mM FeCl_3_ as catalyst. An electrochemical workstation (CHI660E, CH Instruments, Shanghai, China) was used to apply electrical power to initiate the polymerization. After 45 min electrochemical deposition at a constant current density of 1.0 mA/cm^2^, the resultant membrane was thoroughly rinsed with deionized (DI) water.

### 2.2. Membrane Characterizations

#### 2.2.1. Surface Morphology and Chemical Composition

The morphology of the composite membrane was observed through a field emission scanning electron microscope (FE-SEM, S4800, Hitachi, Japan). Surface functional groups and elemental composition were determined with an attenuated total reflectance Fourier transform infrared spectrometer (ATR-FTIR, Nicolet iS10, Thermo Fisher, Waltham, MA, USA) and an X-ray photoelectron spectrometer (XPS, ESCALAB 250Xi, Thermo Scientific, Waltham, MA, USA), respectively.

#### 2.2.2. Membrane Surface Wettability

Membrane surface wettability was assessed in terms of water contact angle using a video-supported contact angle meter (OCA20, Dataphysics, Filedrstadt, Germany) and a sessile-drop method. To measure the water contact angle, a 2 μL water droplet was placed on the surface of a dried membrane. The dynamic variation in the droplet profile on the membrane surface was recorded, and fitting software (SC20, Version 4.3, Dataphysics, Filedrstadt, Germany) was used to calculate the contact angle in each frame. For each membrane, the result was an average of at least six parallel measurements of three separately prepared samples.

#### 2.2.3. Membrane Permeability

A dead-end filtration system equipped with a stirred ultrafiltration cell (Amicon 8010, Merck KGaA, Darmstadt, Germany) was used to determine membrane permeability (m s^−1^ kPa^−1^) with DI water in a constant-pressure manner. Each membrane sample was pre-compacted for 20 min at 20 kPa. Then, the permeability was recorded for 10 min at 8 kPa.

### 2.3. Evaluation of Antifouling Performance

#### 2.3.1. Preparation of Synthetic Foulant Solutions

Considering that hardness ions (i.e., Ca^2+^, Mg^2+^) play a unique bridging role in the development of membrane fouling [37], two types of synthetic foulant solutions were prepared to investigate antifouling behavior with or without the presence of Ca^2+^ and Mg^2+^. As shown in Table 1, sodium alginate (SA, Sigma–Aldrich, St. Louis, MO, USA), humic acid (HA, Sigma-Aldrich, St. Louis, MO, USA), and bovine serum albumin (BSA; ~66 kDa; Sigma–Aldrich, St. Louis, MO, USA) were selected as organic model foulants to represent polysaccharides, natural organic compounds, and proteins in practical wastewater, respectively [9,10,38,39]. CaCl_2_ (dihydrate, AR; Sinopac Chemical Reagents Co., Ltd., Danyang, China) and MgCl_2_ (hexahydrate, AR; Sinopac Chemical Reagents Co., Ltd., St. Louis, MO, USA) were used to provide hardness ions. NaCl (crystal, AR; Sinopac Chemical Reagents Co., Ltd., St. Louis, MO, USA) and NaHCO_3_ (crystal, AR, Sinopac Chemical Reagents Co., Ltd., St. Louis, MO, USA) were used to adjust the ionic strength and pH of the solution, respectively. All chemicals were used as received.

#### 2.3.2. Cross-Flow Filtration System

The antifouling filtration tests were carried out using a cross-flow filtration system, the core unit of which was a cross-flow filtration cell (Figure S1) with an effective membrane filtration area of 1 × 5 cm^2^. To achieve an in situ switching of wettability, a titanium foil was placed opposite to the conductive SSM/PPY(AOT) membrane so that external voltages could be applied to the membrane (as a working electrode) and the titanium foil (as a counter electrode) to initiate wettability switching reactions. Silicone rubber frames were appropriately packed in the cell to prevent leaching. A peristaltic pump (BT100−1L, Longer Pump, Baoding, China) was used to drive feed solution to flow through the membrane. Changes in the transmembrane pressure (TMP) were recorded by an electronic recorder (MIK-R6000C, MEACON, Hangzhou, China).

#### 2.3.3. Antifouling Filtration Tests

To investigate the fouling behaviors of the SSM/PPY(AOT) membrane under different wetting conditions (i.e., hydrophilic in reduction state and hydrophobic in oxidation state; Figure 1), four different filtration operating modes were designed, including (i) an M_R_ mode: filtration and membrane cleaning in reduction state; (ii) an M_O_ mode: filtration and membrane cleaning in oxidation state; (iii) an M_OR_ mode: filtration in oxidation state and membrane cleaning in reduction state; and (iv) an M_RO_ mode: filtration in reduction state and membrane cleaning in oxidation state.

In the test for each mode, a five-cycle filtration operation comprising alternated fouling filtration and membrane cleaning procedures was carried out. In each cycle, a 30 min filtration with the foulant solution was performed. Before and after each filtration with the foulant solution, a 10 min filtration with a 0.1 M NaCl solution was carried out for determination of foulant-free water permeability. When the wettability switching was needed (i.e., in the M_OR_ and M_RO_ modes), a charging operation was conducted at the last minute of the NaCl filtration operation. At the end of each cycle, the membrane was physically rinsed with DI water for 10 min.

## 3. Results and Discussion

### 3.1. Surface Properties of the SSM/PPY(AOT) Membrane

**Figure 2:**
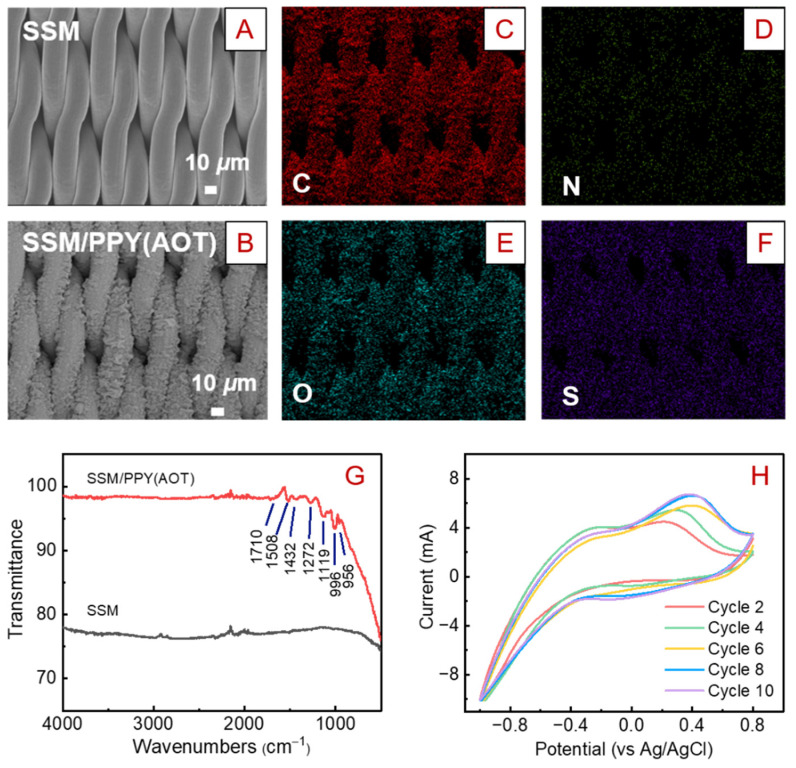
Characterizations of the SSM and SSM/PPY(AOT) membranes. SEM images of the (**A**) SSM and (**B**) SSM/PPY(AOT) membranes. Elemental mappings of the SSM/PPY(AOT) membrane: (**C**) C, (**D**) N, (**E**) O, and (**F**) S. (**G**) FTIR spectra of the SSM and SSM/PPY(AOT) membrane. (**H**) Cyclic voltammetry curves for the SSM/PPY(AOT) membrane.

As shown in Figure 2A, the pristine SSM possesses a woven porous structure. After 45 min electro-polymerization, the surface of the SSM was completely covered by characteristic aggregates of microgranular grains (Figure 2B; see cross-sectional images in Figure S2), which should have been the formed PPY doped with AOT. The resultant SSM/PPY(AOT) composite membrane still maintained the woven porous structure based on visual observation. The addition of the small amount of FeCl_3_ to the electrolyte solution during the polymerization allowed the co-occurrence of electrical and chemical polymerization, and this composite polymerization process could promote the growth of loose and porous PPY [40]. Elemental mapping shows that C, N, O, and S elements are evenly distributed on the membrane surface (Figure 2C–F), further indicating that the AOT-doped PPY was uniformly deposited on the surface of the SSM.

Figure 2G presents the FTIR spectra of the SSM and SSM/PPY(AOT) membrane. Basically, there is no obvious characteristic peak in the SSM. For the SSM/PPY(AOT) membrane, several characteristic peaks were observed, which could verify the success of the PPY(AOT) formation. The peak at 1710 cm^−1^ can be associated with the C=O groups of AOT. The peak relative to PPY ring vibration was observed at 1508 cm^−1^. The peaks at 1432 and 1272 cm^−1^ can be associated with the vibration of C–N in PPY. The peaks at 1119 and 996 cm^−1^ can be attributed to the vibration of S=O in AOT, thus demonstrating the successful doping of AOT in the PPY backbone. These results correspond well with previous studies [41,42,43].

In order to investigate the electrochemical performance of the membrane, the redox switching behavior of the SSM/PPY(AOT) membrane was examined in 0.1 M NaCl at 10 mV s^−1^ using an electrochemical workstation. As shown in Figure 2H, the resultant cyclic voltammetry curves show a pair of redox peaks, with the anodic peak at 0.4 V and the cathodic peak at −0.25 V, which are associated with Na^+^ exiting and entering the polymer matrix, respectively [44]. Accordingly, 0.2 V and −0.8 V were chosen as the oxidation and reduction potentials for the SSM/PPY(AOT) membrane, respectively. Additionally, the current–time curves for the oxidation and reduction processes suggest that the current changes tended to be stable after 60 s (Figure S3). Therefore, for the purpose of wettability switching via electrochemical reactions, a >60 s charging operation would be sufficient to complete the oxidation or reduction of the SSM/PPY(AOT) membrane.

### 3.2. Reversible Wettability Switching

**Figure 3:**
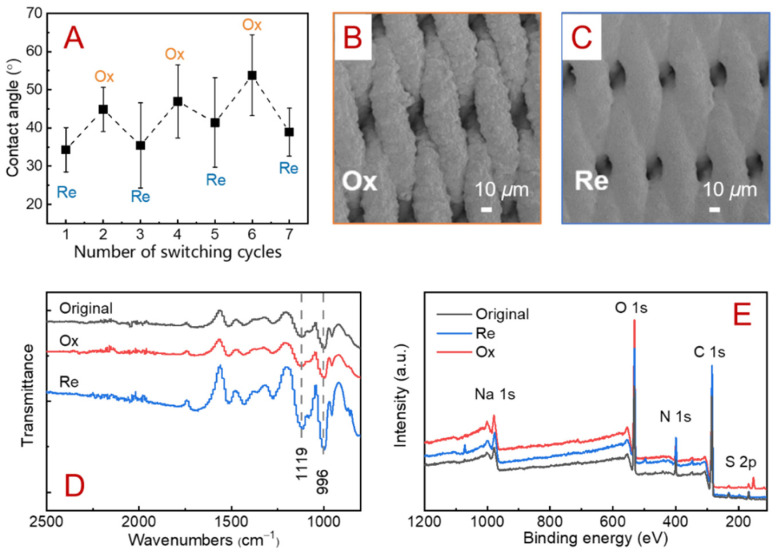
(**A**) Variation in measured water contact angles as the membrane switched between its oxidation (Ox) and reduction (Re) states. SEM views of the membrane in its (**B**) oxidation and (**C**) reduction states. (**D**) FTIR spectra and (**E**) XPS survey scans of the SSM/PPY(AOT) membrane in its original, oxidation, and reduction states.

In order to examine the wettability switching behavior of the membrane under electrical stimulation, water contact angles on the SSM/PPY(AOT) membrane surface were measured. As shown in Figure 3A, the membrane had smaller contact angles in the reduction state than in the oxidation state, indicating that the membrane was more hydrophilic in the reduction state. In general, the membrane can switch to a relatively hydrophobic state after a 1 min oxidation treatment, and the contact angles normally could increase by ~10–15°. In addition, the wettability switch between hydrophilic and relatively hydrophobic states can be repeated many times.

Such reversible wettability switching can be attributed to the reversible changes in both membrane morphology and surface chemistry. Comparing the SEM images in Figure 3B,C, it was observed that the local membrane surface became rougher in the oxidation state and smoother in the reduction state. During the redox process for the SSM/PPY(AOT) membrane, small anions usually leave or return to the main chain of PPY, while the AOT ions have difficulty in moving due to their relatively larger volume. Instead, Na^+^ would enter or be expelled from the main chain of PPY to balance the charge, resulting in structural changes to the PPY(AOT). Therefore, as observed in Figure 3B,C, the SSM/PPY(AOT) membrane exhibited higher micro-roughness in the oxidization state than in the reduction state. Such increased roughness could inhibit complete contact between water and membrane surfaces, resulting in increased water contact angles [11,39,45].

Meanwhile, the changes in surface functional groups and elemental compositions in the redox process were investigated by FTIR and XPS. As shown in Figure 3D, the peaks at 1119 and 996 cm^−1^ relative to the S=O vibration in AOT became weaker in the oxidation state than in the reduction state, indicating that the –SO^3−^ group was more exposed to the outer surface in the reduction state (Figure 1B). These results correspond well with those of a previous study [43].

The surface chemical compositions of the membrane in its original, oxidation, and reduction states were further assessed by XPS (Figure 3E). In the wide-scan spectra of the SSM/PPY(AOT) membrane in different states, C 1s, O 1s, N 1s, Na 1s, and S 2P signals were detected. Compared with the intensity in the original state, the Na 1s intensity in the reduction state was relatively higher, and the contents of Na on the surface also increased from 0.16% to 1.12%. However, in the oxidation state, the peak intensity and content of Na on the membrane surface were much lower than in the reduction state. This suggested that Na^+^ entered the main chain of PPY when the membrane was reduced and left when the membrane was oxidized. These data verified that in the reduction state the electrostatic attractions between the sulfonic groups of AOT and the PPY backbone were weakened, so that more sulfonic groups were exposed to the membrane surface and attracted Na+ to balance the charge. These results are consistent with previous observations [43].

### 3.3. Antifouling Behaviors in Different Operating Modes

A series of filtration tests in different modes (i.e., M_R_, M_O_, M_RO_, and M_OR_) were carried out to evaluate the antifouling performance of the SSM/PPY(AOT) membrane. In each filtration test, fouling filtration and membrane cleaning procedures were alternately performed. Synthetic foulant solutions (with or without Ca^2+^ and Mg^2+^; Table 1) were used as feed solutions to induce membrane fouling. In the M_R_ mode, the SSM/PPY(AOT) membrane was maintained in the reduction state for all the fouling filtration and cleaning processes. In the M_O_ mode, the membrane was maintained in the oxidation state for all the processes. In the M_OR_ mode, the fouling filtration was conducted in the oxidation state while the cleaning operation was conducted after the membrane was switched to its reduction state. In the M_RO_ mode, the fouling filtration and cleaning operation were performed in the reduction and oxidation states, respectively.

**Figure 4:**
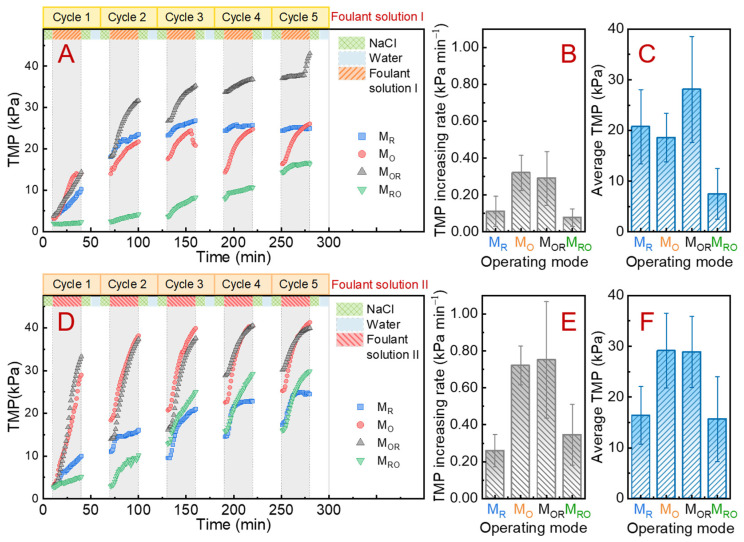
Comparison of antifouling performance in terms of (**A**) TMP variation, (**B**) TMP increasing rate, and (**C**) average TMP among the different operating modes (i.e., M_R_, M_O_, M_OR_, and M_RO_) with the synthetic foulant solution I (Table 1). Comparison of antifouling performance in terms of (**D**) TMP variation, (**E**) TMP increasing rate, and (**F**) average TMP among the different operating modes with the synthetic foulant solution II (Table 1).

As presented in Figure 4A,C, for all the operating modes, the TMP gradually increased as the fouling filtration was started, indicating an increase in membrane filtration resistance due to membrane fouling. The average TMP increasing rates and average TMPs during the fouling filtration process were calculated (Figure 4B,C,E,F). Apparently, for both the tests with foulant solutions I and II, the average TMP increasing rates in the M_O_ and M_OR_ modes (hydrophobic state for fouling filtration) were significantly higher than those in the M_R_ and M_RO_ modes (hydrophilic state for fouling filtration). This demonstrates that the hydrophilic reduction state of the SSM/PPY(AOT) membrane is more resistant to fouling. However, in the relatively hydrophobic oxidation state, foulants tend to accumulate on the membrane surface, probably due to enhanced hydrophobic force [46].

After the fouling filtration in each cycle, a 10 min physical cleaning could make the TMP fall back to a certain extent. Theoretically, it was expected that a hydrophobic membrane surface with lower surface energy would be relatively incapable of capturing foulants. So, it was expected that the SSM/PPY(AOT) membrane would perform better in antifouling in the M_RO_ mode, where the hydrophilic state during fouling filtration could resist foulant accumulation and the hydrophobic state during cleaning could facilitate the detachment of adsorbed foulants. As shown in Figure 4A–C (tests without Ca^2+^ and Mg^2+^), the membrane did indeed maintain lower TMPs and TMP increasing rates in the M_RO_ mode than in the M_R_ mode. This demonstrates the importance of wettability switching during membrane filtration and suggests the potential advantage of this SSM/PPY(AOT) membrane for practical applications. In addition, the TMP drop at the end of the third cycle for the M_O_ mode in Figure 4A was most likely due to accidental air leakage, which did not significantly influence the comparisons.

When Ca^2+^ and Mg^2+^ were included in the foulant solution, higher TMPs and TMP increasing rates were observed (Figure 4D–F). This indicated that the rate of membrane fouling during filtration was accelerated. The aggravation of membrane fouling could be due to complexation among the sulfonic acid groups in AOT (especially in reduction state), Ca^2+^/Mg^2+^, and foulants. It is noteworthy that the membrane still maintained lower TMPs in the first two cycles in the M_RO_ mode than in the M_R_ mode. However, it may be that the complexed Ca^2+^/Mg^2+^ in the first two cycles hindered the wettability switching process, and the charging process may also have affected the surface properties of the membrane, its antifouling capability having weakened in the following three cycles in the M_RO_ mode.

**Figure 5:**
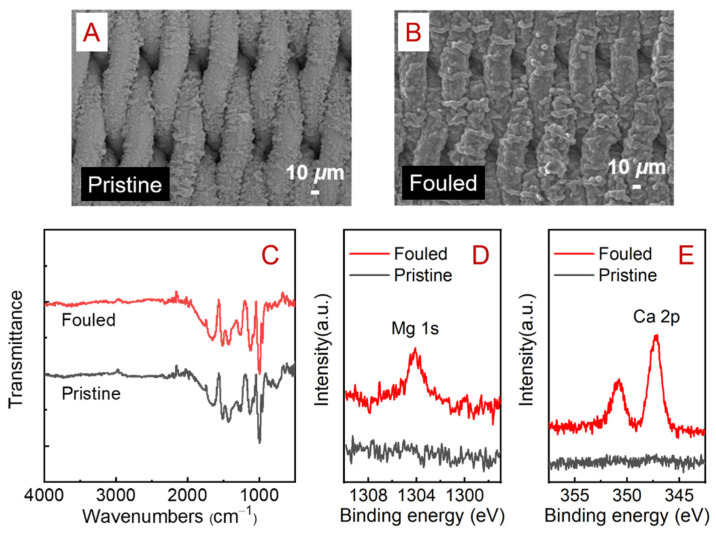
SEM views of the (**A**) pristine and (**B**) fouled SSM/PPY(AOT) membranes operated in the M_RO_ mode in the antifouling filtration experiments with foulant solution II. (**C**) FTIR spectra and (**D**,**E**) XPS analyses of the pristine and fouled membranes.

Figure 5A,B show the SEM views of the pristine and fouled membrane surfaces in the M_RO_ mode with the foulant solution II. Compared with the fouling status of the fouled membranes in the M_R_, M_O_, and M_OR_ modes (Figure S4), the fouling status in the M_RO_ was much less severe. In the M_R_, M_O_, and M_OR_ modes, the original loose and porous PPY structure was blocked by foulants, and some large foulant particles also appeared on the membrane surface. These would increase filtration resistance, resulting in higher TMPs. By contrast, far fewer foulants were observed on the fouled membrane in the M_RO_ mode, and there was no significant change in the FTIR spectra (Figure 5C). This indicates that the SSM/PPY(AOT) membrane performs better in antifouling in the M_RO_ mode. However, as shown in Figure 5D,E, the signals associated with Ca and Mg in the XPS spectra for the fouled membrane surface (M_RO_) significantly increased, suggesting complexation between Ca^2+^/Mg^2+^ and sulfonic acid groups on the membrane surface.

## 4. Conclusions

A conductive SSM/PPY(AOT) composite membrane with reversible wettability was prepared by electro-polymerization. The wettability of the membrane could be reversibly switched via electrical stimulation. In the reduction state, the sulfonic acid groups of AOT were more exposed to the membrane surface, rendering the surface more hydrophilic. In the oxidation state, the aliphatic chains of AOT were more exposed to the surface and thus more hydrophobic. The fouling filtration experiments verified that the membrane is more resistant to fouling in the hydrophilic state during fouling and easier to clean in the hydrophobic state. Additionally, Ca^2+^ and Mg^2+^ would complex with foulants and aggravate membrane fouling. Overall, this study demonstrates the importance of wettability switching during membrane filtration and suggests the potential advantage for practical applications of the SSM/PPY(AOT) membrane.

## Figures and Tables

**Table 1:** Chemistry of two synthetic foulant solutions for antifouling filtration tests.

Items	Component	Concentration	
		Foulant Solution I	Foulant Solution II
Organic (mg L^−1^)	Sodium alginate (SA)	25	25
	Humic acid (HA)	15	15
	Bovine serum albumin (BSA)	8	8
Inorganic (mM)	CaCl_2_	0	1
	MgCl_2_	0	0.5
	NaHCO_3_	2	2
	NaCl	18	9

## Data Availability

Not applicable.

## Supplementary Materials

The following supporting information can be downloaded at: https://www.mdpi.com/article/10.3390/membranes12060626/s1, Figure S1: Configuration diagram of the cross-flow filtration cell used in the antifouling filtration experiments; Figure S2: SEM images of cross sections of the (A) SSM and (B) SSM/PPY(AOT) membranes; Figure S3: Current–time curves for the oxidation and reduction processes for the SSM/PPY(AOT) membrane; Figure S4: SEM views of the fouled membrane in the M_R_ mode, the fouled membrane in the M_O_ mode, and the fouled membrane in the M_OR_ mode.
