# Supplementary material for: Antifouling Conductive Composite Membrane with Reversible Wettability for Wastewater Treatment
Source: Membranes (Basel). 2022 Jun 16;12(6):626. doi: 10.3390/membranes12060626 (PMC9231251; doi:10.3390/membranes12060626)
Supplement: Supplementary file 1 [file membranes-12-00626-s001.zip › membranes-1774668-supplementary.pdf]

Supporting Information

Antifouling conductive composite membrane with reversible wettability for wastewater treatment

Yi Li[1,2], Ruonan Gao[1,2], Jianwen Zhang[1,2], Yue Zhang[1,2] and Shuai Liang[1,2,*]

[1] Beijing Key Lab for Source Control Technology of Water Pollution, College of Environmental Science and Engineering, Beijing Forestry University, Beijing 100083, China

[2] Engineering Research Center for Water Pollution Source Control & Eco-remediation, College of Environ-mental Science and Engineering, Beijing Forestry University, Beijing 100083, China

*Corresponding author. Tel.: +86 (10) 6233 6675; fax: +86 (10) 6233 6596.

E-mail: shuai_liang@bjfu.edu.cn

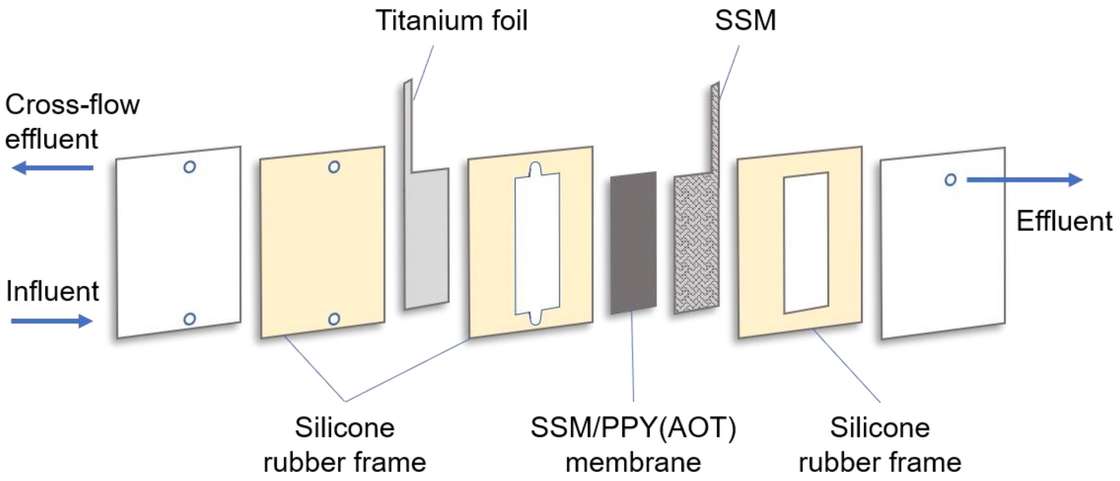

Figure S1. Configuration diagram of the cross-flow filtration cell used in the antifouling filtration experiments.

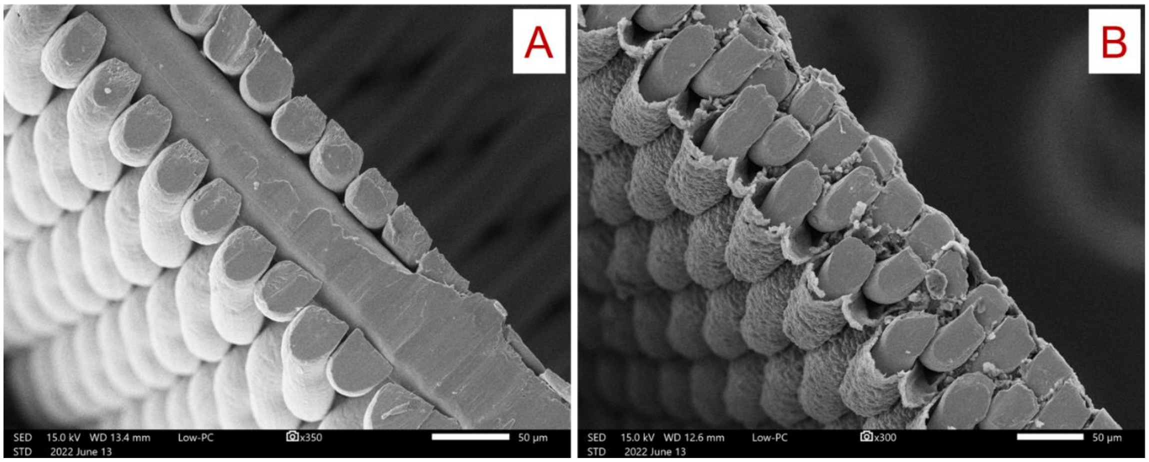

Figure S2. SEM images of cross sections of the (A) SSM and (B) SSM/PPY(AOT) membrane.

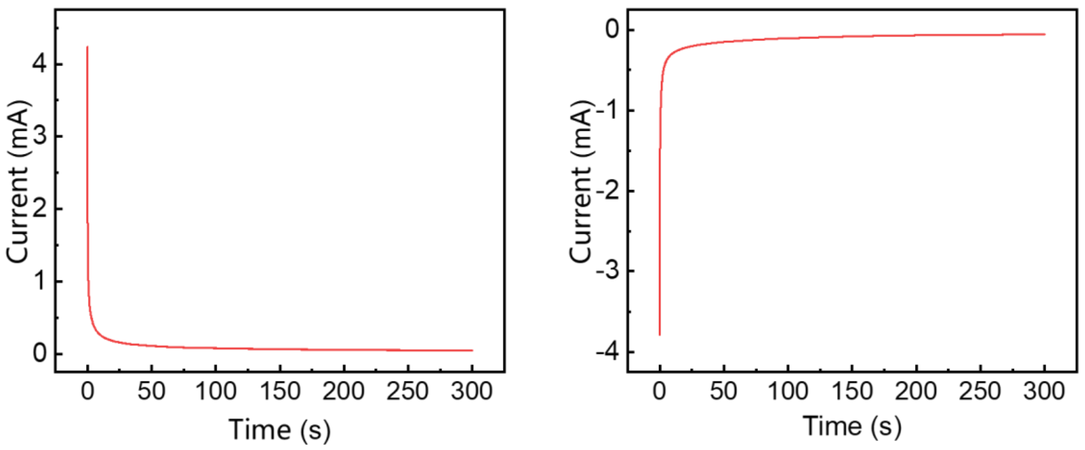

Figure S3. Current-time curves of the oxidation and reduction processes for the SSM/PPY(AOT) membrane.

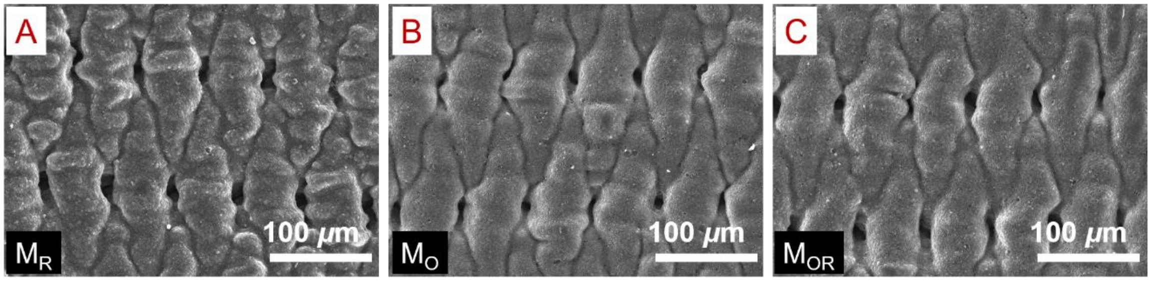

Figure S4. SEM views of the (A) fouled membrane in the M_R mode, (B) fouled membrane in the M_O mode, and (C) fouled membrane in the M_{OR} mode.
